# FMF Is Associated With a Wide Spectrum of MHC Class I- and Allied SpA Disorders but Not With Classical MHC Class II-Associated Autoimmune Disease: Insights From a Large Cohort Study

**DOI:** 10.3389/fimmu.2019.02733

**Published:** 2019-11-26

**Authors:** Abdulla Watad, Nicola Luigi Bragazzi, Mohammad Adawi, Yehuda Shoenfeld, Doron Comaneshter, Arnon D. Cohen, Dennis McGonagle, Howard Amital

**Affiliations:** ^1^Department of Medicine B and Zabludowicz Center for Autoimmune Diseases, Sheba Medical Center, Ramat Gan, Israel; ^2^Sackler Faculty of Medicine, Tel-Aviv University, Tel-Aviv, Israel; ^3^Section of Musculoskeletal Disease, NIHR Leeds Musculoskeletal Biomedical Research Unit, Leeds Institute of Molecular Medicine, University of Leeds, Chapel Allerton Hospital, Leeds, United Kingdom; ^4^Department of Health Sciences (DISSAL), Postgraduate School of Public Health, University of Genoa, Genoa, Italy; ^5^Laboratory for Industrial and Applied Mathematics, Department of Mathematics and Statistics, York University, Toronto, ON, Canada; ^6^Azrieli Faculty of Medicine, Padeh and Ziv Hospitals, Bar-Ilan University, Ramat Gan, Israel; ^7^Sechenov First Moscow State Medical University of the Ministry of Health of the Russian Federation (Sechenov University), Moscow, Russia; ^8^Chief Physician's Office, Clalit Health Services, Tel-Aviv, Israel; ^9^Faculty of Health Sciences, Siaal Research Center for Family Medicine and Primary Care, Ben Gurion University of the Negev, Beer Sheva, Israel

**Keywords:** Familial Mediterranean fever, spondyloarthritis, Crohn's disease, ulcerative colitis, MHC-I

## Abstract

**Objectives:** To test the hypothesis that familial Mediterranean fever (FMF)-associated autoinflammation may exaggerate the tendency toward adaptive immunopathology or spondyloarthritis (SpA)-associated disorders including major histocompatibility complex (MHC) class I associated disorders but not classical MHC class II-associated disorders that exhibit transplacental autoimmunity including myasthenia gravis and pemphigus.

**Methods:** Seven thousand seven hundred forty-seven FMF patients and 10,080 age- and sex-matched controls in the Clalit Health Services medical database were identified and compared in terms of prevalence of SpA-associated disorders. We also evaluated four classical and strong MHC class II-associated disorders, namely, pemphigus vulgaris, myasthenia gravis, sarcoidosis, and pernicious anemia, to ascertain whether such associations with SpA-spectrum disease were specific or merely reflected the non-specific consequences of innate immune system activation on driving divergent types of immunity. The diagnosis of FMF was based on the medical records and not genetically proven.

**Results:** FMF showed a strong association with MHC class I-related diseases: odds ratio (OR) of 28.58 [95% confidence interval (95% CI), 6.93–117.87; *p* < 0.0001] for Behçet's disease, OR of 10.33 (95% CI, 4.09–26.09; *p* < 0.0001) for ankylosing spondylitis, and OR of 1.67 (95% CI, 1.19–2.33; *p* = 0.0029) for psoriasis. For weakly MHC class I-linked diseases, an OR of 3.76 (95% CI, 2.48–5.69; *p* < 0.0001) for Crohn's disease and OR of 2.64 (95% CI, 1.52–4.56; *p* = 0.0005) for ulcerative colitis were found. No association was found between FMF and the four MHC class II-associated autoimmune disorders.

**Conclusion:** FMF patients are associated with increased risk of SpA-related disease diagnosis including MHC-I-opathies but not MHC-II-associated autoimmune diseases, suggesting that tissue-specific dysregulation of innate immunity share between FMF and SpA spectrum disorders may drive adaptive immune MHC class I-associated conditions.

## Introduction

Familial Mediterranean fever (FMF) is one of the paradigmatic hereditary autoinflammatory disorders, caused by point mutations, either non-sense or missense, in the Mediterranean fever (*MEFV*) gene, generally inherited in an autosomal recessive way (1). It is characterized by brief, painful, recurrent episodes of skin rash (usually, erysipelas-like erythema), peritonitis, pleuritis, synovitis, arthritis, and, rarely, pericarditis and meningitis, accompanied by fever.

Historically, Behcet's disease (BD), Crohn's disease (CD), ulcerative colitis (UC), psoriasis, and ankylosing spondylitis (AS) were classified under the spondyloarthritis (SpA) umbrella term (2). FMF and the SpA group of disorders share an association with disease localization to site of physical stress or microdamage with innate immune activation at such sites as a primary driver of immunopathology (3, 4).

With the development of the immunological disease continuum model of inflammation against self, these disorders were placed as intermediates between autoinflammation and autoimmune boundary of self-directed inflammation (5). Recently, we pointed out how BD, psoriasis, anterior uveitis, and AS fitted under the unifying umbrella term of “MHC-I-opathies,” due to shared immunopathogenetic mechanisms, a common association with tissue-specific microdamage, disturbed and impaired barrier function of skin, mouth and gut, and role of innate lymphoid cells surveillance in the context of IL23–IL17 axis-related genetic polymorphisms (5). Although CD and UC share many of these features, specific major histocompatibility complex (MHC) class I associations at the population level are very weak, suggesting a greater contribution of innate immunity at the population level (5, 6).

Several case series and small studies have reported potential associations between FMF and SpA-related diseases and indeed other rheumatic disorders including rheumatoid arthritis (RA) (7–9). However, the RA association with *MEFV*-related mutations may specifically be with antibody negative or “autoinflammatory-type RA” (5). We utilized a large cohort of FMF cases where the disease is known to represent a tissue-specific innate immunopathology (10). We then looked at the association between the “MHC-I-opathy”-related conditions including AS, psoriasis, and BD and, at the population level non-MHC class I SpA-associated diseases, namely, CD and UC, all of which are also associated with tissue-specific dysregulation as key pathological drivers. We also looked at MHC class II-associated diseases, especially autoantibody-associated diseases to ascertain whether such associations with SpA spectrum disease were specific or merely reflected the non-specific consequences of innate immune system activation on driving divergent types of immunity.

## Materials and Methods

### Ethical Approval

The study protocol of the current investigation was approved by the Ethical Committee of the Clalit Health Services, located at the Soroka Medical Center, Beer-Sheva, Israel.

### Study Population

Data were collected from the Clalit Health Services Database, the largest state-mandated health service organization in Israel. The data undergo an extensive series of cross-check and quality verification by comparing diagnoses from various sources. The validity of the data was verified to be high, as shown in previously published studies (11–14). Wide-scale epidemiological studies can be conducted in real time on heterogeneous groups using advanced, massive data-mining techniques from the database.

The FMF patients were identified based on at least two diagnosis of FMF in their medical records given by general practitioner, primary care physician, or a specialist. FMF has ethnic predilection to nations of the Mediterranean region, being frequently observed among Turkish, Jewish, Arabic, and Armenian communities. The Clalit Health Services database comprises ~7,700 patients. All FMF patients detected in the Clalit Health Services database were considered eligible and, as such, were enrolled in the present study. The control group involved randomly selected Clalit Health Services enrollees excluding the patients with an established diagnosis of FMF. Controls were age- and sex-matched to cases.

Similarly to FMF, the diagnosis of SpA-related diseases (CD, UC, psoriasis, BD, and AS), included patients with such certified diagnosis in their medical records at least twice as entered by specialists in the Clalit Health Services registry. We also selected MHC class II and autoantibody-associated diseases (pernicious anemia, myasthenia gravis, and pemphigus vulgaris) and the MHC class II non-autoantibody-associated disease (sarcoidosis) to evaluate whether FMF was non-specifically associated with an array of inflammatory disorders or linked to diseases where tissue-specific innate immune dysregulation is a key early feature. Data collected from the Clalit Health Services database included relevant sociodemographic and clinical information such as age, gender, socioeconomic status (SES), body mass index (BMI), and smoking status. More in detail, SES was defined according to the poverty index of the member's residence area. Briefly, poverty index is computed from several parameters including household income, education, and other factors, which are clustered together and ranked. The composite index ranged from 1 to 20 with 1 as the lowest SES.

### Statistical Analyses

Before processing the data, they were visually inspected for potential outliers. Normality of data distribution was verified applying the D'Agostino-Pearson *omnibus* test. Continuous variables were computed as mean ± standard deviation, whereas categorical parameters were expressed as percentages.

The occurrence of FMF and of SpA spectrum disorders was compared between FMF patients and controls in the selected study sample. The chi-squared test was used to assess the distribution of categorical variables, while the *t*-test and ANOVA were applied for continuous variables. Moreover, the association between FMF and SpA-associated disorders was assessed using a multivariate logistic regression model and a multivariate Cox proportional-hazards regression model, both adjusted for possible confounding factors.

Analyses regarding survival rates were performed using the Kaplan–Meier curves, the log-rank test, and the multivariate Cox proportional-hazards method to detect factors associated with increased all-cause risk mortality, with adjustment for risk factors where appropriate.

All statistical analyses were performed using the commercial software “Statistical Package for the Social Sciences” for Windows (SPSS version 24.0, SPSS Inc., IBM, USA). Graphs were generated utilizing the commercial software MedCalc version 17.9.7 for Windows (MedCalc Software bvba, Ostend, Belgium; http://www.medcalc.org; 2017). For all analyses, figures with *p* < 0.05 were considered statistically significant.

## Results

### Basic Characteristics of the Study Population

The current study included 7,747 FMF patients and 10,080 age- and sex-matched controls. The two groups did not differ in terms of BMI, while they differed in terms of SES (*p* = 0.0200), with low and medium strata being overrepresented among FMF patients. Smokers were more present among FMF patients (2,412, 31.1%, vs. 2,588, 25.7%, *p* < 0.0001).

### The Proportion of the Different SpA-Related Disorders and MHC-I-Opathies in FMF and Controls

**Figure 1:**
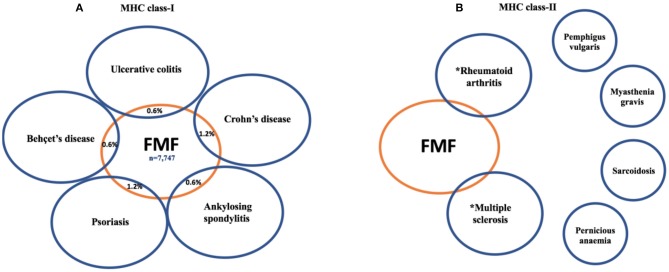
The link between familial Mediterranean fever (FMF) patients and major histocompatibility complex (MHC) class I-associated disorders **(A)** compared to MHC class II-associated disorders **(B)**. Unlike the MHC class I-associated disorders where a link with FMF is consistently reported the situation for MHC class II diseases is less clear. FMF has been linked to RA, but ~30% of RA cases are seronegative, and some of these may be innate immune mediated (15). Weak associations between FMF and MS have been reported, but putative-disease-associated autoantibodies remain controversial. The other classical autoimmune diseases with MHC class II and autoantibody associations have not been linked to *MEFV* mutations. ^*^These findings are based on the references (16, 17).

The SpA spectrum disorder diagnosis was significantly higher in FMF patients compared with controls (326 cases, 4.2%, vs. 129 cases, 1.3%, *p* < 0.0001) (Figure 1A). For further details, the reader is referred to Figure 1A and Table 1.

**Table 1:** Overall population, familiar Mediterranean fever (FMF) patients (cases) and age- and sex-matched controls—basic characteristics.

**Characteristic**	**All population (*n* = 17,827)**	**Controls without FMF (*n* = 10,080)**	**FMF patients (*n* = 7,747)**	**Statistical significance (*p* value)**
Age (mean ± SD)	38.43 ± 19.62	37.69 ± 19.55	39.38 ± 19.68	NS
Age at diagnosis (mean ± SD)	26.41 ± 18.41	25.67 ± 18.35	27.37 ± 18.45	NS
Gender (female; %)	9,000 (50.5%)	5,121 (50.8%)	3,879 (50.1%)	NS
BMI (mean ± SD)	24.81 ± 63.91	24.42 ± 50.61	25.30 ± 77.41	NS
SES (*n*; %)^a^				*p* = 0.02
Low	8,370 (50.6%)	4,729 (50.3%)	3,641 (51.1%)	
Medium	5,609 (33.9%)	3,153 (33.5%)	2,455 (34.5%)	
High	2,548 (15.4%)	1,524 (16.2%)	1,024 (14.4%)	
Smoking (*n*; %)	5,000 (28.0%)	2,588 (25.7%)	2,412 (31.1%)	<0.0001
SpA-related disorders	455 (2.6%)	129 (1.3%)	326 (4.2%)	<0.0001
Psoriasis	156 (0.9%)	66 (0.7%)	90 (1.2%)	0.0003
Behçet's disease	51 (0.3%)	2 (0.02%)	49 (0.6%)	<0.0001
Ankylosing spondylitis	53 (0.3%)	6 (0.1%)	47 (0.6%)	<0.0001
Crohn's disease	130 (0.7%)	35 (0.3%)	95 (1.2%)	<0.0001
Ulcerative colitis	65 (0.4%)	20 (0.2%)	45 (0.6%)	<0.0001
All-cause mortality (*n*; %)	707 (4.0%)	341 (3.4%)	366 (4.7%)	<0.0001

a *Data available for 92.7% of the population*.

### The Risk of SpA-Related Disorder and MHC-I-Opathy Diagnosis in FMF Patients

At the univariate analysis, FMF had a 3.2-fold higher chance of being diagnosed with a SpA-related disorders compared with controls. After adjusting for confounders, FMF patients had an odds ratio (OR) of 3.27 [95% confidence interval (95% CI), 2.62–4.10; *p* < 0.0001] of developing SpA-related disorder.

More specifically, an increased risk of an OR of 28.58 (95% CI, 6.93–117.87; *p* < 0.0001) for BD, an OR of 10.33 (95% CI, 4.09–26.09; *p* < 0.0001) for AS, and an OR of 1.67 (95% CI, 1.19–2.33; *p* = 0.0029) for psoriasis, an OR of 3.76 (95% CI, 2.48–5.69; *p* < 0.0001) for CD, and an OR of 2.64 (95% CI, 1.52–4.56; *p* = 0.0005) for UC was found.

The multivariate Cox proportional-hazards regression analysis confirmed this link: a hazard ratio (HR) of 27.92 (95% CI, 6.77–115.13; *p* < 0.0001) for BD and HR of 9.72 (95% CI, 3.85–24.55; *p* < 0.0001) for AS, HR of 1.62 (95% CI, 1.16–2.26; *p* = 0.0046) for psoriasis, HR of 3.68 (95% CI, 2.43–5.57; *p* < 0.0001) for CD, and HR of 2.52 (95% CI, 1.46–4.36; *p* = 0.0009) for UC was found.

### A Lack of Association Between FMF and Strong MHC Class II-Associated Diseases

To assess whether FMF was associated with all autoimmune diseases regardless of the mechanoinflammatory SpA environment (18) and MHC class I components, we selected four strongly associated MHC class II-related diseases, and no association was found with FMF (Figure 1B). Indeed, FMF patients had an OR of 1.67 (95% CI, 0.28–10.05; *p* = 0.5763) for pemphigus vulgaris and an OR of 1.80 (95% CI, 0.30–10.84; *p* = 0.5208) for myasthenia gravis, noting that both these diseases may exhibit transplacental autoimmunity. Likewise, an OR of 1.91 (95% CI, 0.86–4.21; *p* = 0.1109) for sarcoidosis, and an OR of 0.65 (95% CI, 0.19–2.23; *p* = 0.4896) for pernicious anemia was computed.

### The Impact of MHC-I-Opathy Diagnosis on the Mortality of FMF Patients

**Figure 2:**
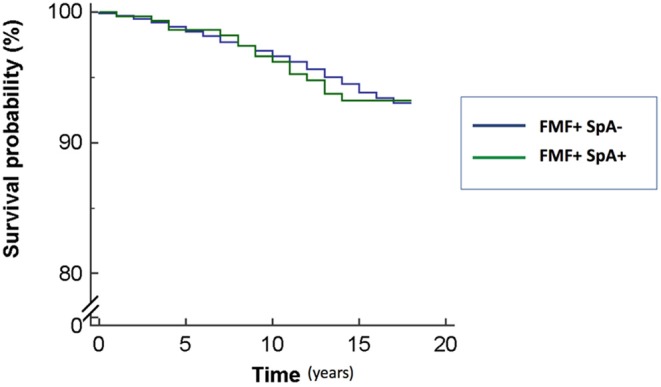
The impact of the presence of spondyloarthritis (SpA) diagnosis on familial Mediterranean fever (FMF) patients' survival.

All-cause mortality was statistically higher in FMF patients than controls (366 deaths, 4.7%, vs. 341 deaths, 3.4%, *p* < 0.0001). At the Cox multivariate survival analysis, SpA-related disorder or MHC-I-opathy diagnosis in FMF patients was not associated with increased all-cause mortality with HR of 0.96 (95% CI, 0.55–1.68; 0.8800) (Figure 2). However, stratifying according to the specific SpA-related disorder, FMF patients with CD had a risk of death of 2.32 (95% CI, 1.09–4.93; *p* = 0.0291) compared to those without CD. Further details are reported in Table 2.

**Table 2:** The impact of different spondyloarthritis (SpA)-related disorders including MHC-I-opathies on the mortality of familial Mediterranean fever (FMF) patients.

**Variable**	**HR**	**95% CI**	***p* value**
SpA-related disorders	0.96	0.55–1.68	0.8800
Behçet's disease	0.44	0.06–3.14	0.4123
Psoriasis	0.72	0.23–2.25	0.5686
Ankylosing spondylitis	1.00	1.00–1.00	0.9561
Crohn's disease	2.32	1.09–4.93	**0.0291**
Ulcerative colitis	1.06	0.26–4.25	0.9393

*Bold value is statistically significant*.

## Discussion

To the best of our knowledge, the current study is the first to systematically explore the association between various SpA-associated diseases (CD, UC, BD, psoriasis, and AS) and also classical autoimmune diseases and FMF. It offers a robust epidemiological evidence that corroborates the concept of MHC-I-opathies and suggests that FMF may share some common mechanisms and pathways. Specifically, the stronger association between FMF and BD and AS and psoriasis points toward secondary adaptive immune activation at sites of *MEFV*-related tissue-specific dysregulation. This association extends to inflammatory bowel disease (IBD) including CD and UC that form part of the SpA spectrum. Furthermore, a lack of association between FMF and those diseases with strong MHC class II associations was observed supporting the concept that these latter disorders are often exclusively linked to central tolerance failure; hence, the impact of *MEFV* mutations is much less or non-existent.

With respect to BD, single nucleotide polymorphisms including *ERAP1, IL23R, IL10*, and *MEFV* variations confirm shared susceptibility genes and inflammatory pathways with SpA-related disorders (19). ERAP1, IL23R, and different MHC class I associations have also been reported for AS, psoriasis, and uveitis (20). We previously reported that the proportion of FMF in patients with BD is significantly higher (5.83 vs. 0.23%, respectively, *p* < 0.001) (21).

Regarding FMF and AS, a meta-analysis (22) found that *MEFV* M694V mutation has a role in susceptibility to AS, with a pooled OR of 3.33 (95% CI, 2.13–5.21). A recent review of the literature (23) reported a highly variable prevalence of AS among FMF patients, ranging from 0.75% (24) to 100% (25), even though an overall significant association between *MEFV* gene mutations and AS has been documented. Concerning psoriasis, there is dearth of evidences, besides anecdotal reports of an association with FMF (8). A study with a sample of 351 FMF found an increased frequency of psoriasis (3.7%) compared to the normal population (26).

A few small studies have investigated the link between FMF and IBD and provided contrasting findings. For instance, Fidder et al. (27) found that the co-occurrence of FMF and CD is characterized by unique clinical features. By means of an extensive computerized search, the authors were able to identify seven patients with concomitant CD and FMF, which is more than the expected prevalence rate in the general population (*p* = 0.03) (27). In particular, CD presented at a significantly later age of incidence in the FMF-CD group (40.6 ± 10.0 years vs. 26.2 ± 11.4 years; *p* < 0.004), whereas FMF was characterized by a higher attack frequency (*p* < 0.05) and increased prevalence of amyloidosis (*p* < 0.02). On the other hand, other studies (28) could not detect any statistically significant association between FMF gene mutations and IBD phenotypic characteristics. Akyuz et al. (29) found a higher overall *MEFV* variation frequency in the IBD (25.5%) patients compared with controls (9.9%, *p* = 0.006). These findings suggest that *MEFV* variations may represent an additional susceptibility factor for IBD, especially if the carrier rate is high.

We found no association between FMF and four MHC class II-associated disorders, namely, pemphigus vulgaris, myasthenia gravis, sarcoidosis, and pernicious anemia. Collectively, these disorders are strongly linked to CD4 T-cell cell immunopathology including follicular helper T-cell germinal center support for autoantibody formation and Th1-type T-cells responses for delayed type IV immune hypersensitivity reactions in sarcoidosis. We included two autoimmune disorders that show transplacental transmission, indicating that pathogenic autoantibodies are a *sine qua non* for disease expression and also suggesting that a role for physically stressed target tissues does not play a role in disease onset, unlike SpA. However, some MHC class II association diseases including rheumatoid arthritis (RA) and multiple sclerosis (MS) were previously reported to be linked to FMF (16, 17). Nevertheless, it is entirely possible that the *MEFV* association with RA is in autoantibody-negative disease or an autoinflammatory variant of disease (15). Previous studies have also shown a link between MS and FMF and reported that homozygosity for the M694V *MEFV* mutation may aggravate the phenotype of MS (16). Although MS is autoimmune in nature, there is plentiful evidence for tissue-specific neuronal damage or degeneration contributing to the phenotype, but this is controversial (30).

Several plausible mechanisms can be behind the link between FMF and SpA-related disorders and MHC-I-opathy. First, these disorders share key features with FMF such as disease localization to sites of mechanical stress, either at barrier surfaces or internal sites. Furthermore, there is an excessive response to normal levels of stress occurring at barrier surfaces or at sites of microdamage, which lead to activations of innate immunity components and in later phases of the adaptive immunity and tissue target damage (20). Furthermore, *MEFV* mutations have been reported by some case reports to be involved also in some SpA-related disorders, mainly those named “MHC-I-opathies” such as BD, AS, and psoriasis (22, 31, 32).

It is generally held that anti-IL-1 therapy is not effective for SpA, which is largely based on a proof of concept study in a group of patients that generally had normal CRP values—a biomarker, that when elevated, predicts response to biological therapy in SpA (33). IL-1 pathway SNPs have been linked to AS (34), and another study showed that the IL-1 antagonist is moderately effective in controlling the clinical manifestations of AS (35). It would be interesting to ascertain whether subjects with resistant AS and SpA showed responses with full IL-1 blockade with monoclonal antibody therapy against IL-1 in populations with a high carriage rate of the *MEFV* gene.

Our study has some limitations, especially the lack of some genetic and clinical characterization of the FMF patients in terms of *MEFV* variations, clinical symptoms, and severity of disease, which might potentially impact on disease course and its association with other comorbidities. The impact of the heterozygous carriage in asymptomatic subjects without FMF and its risk for MHC-I-opathies needs consideration. Given the link between FMF and pyrin inflammasome pathway activation and dysregulated production of IL-1beta in particular, these findings might point toward a role for IL-1 pathway blockade in SpA subjects in populations that have FMF or carry *MEFV* heterozygous mutations, but this needs further study.

In conclusion, our study showed a statistically significant association between FMF and the risk of developing SpA group of diseases including those termed as MHC-I-opathy but not those with a strong MHC class II association (36). This could have important practical implications for the elucidation of etiopathogenesis of MHC-I-opathy disorders and may pave the way for future therapeutic strategies in these cases.

## Data Availability Statement

The datasets generated for this study are available on request to the corresponding authors.

## Ethics Statement

The studies involving human participants were reviewed and approved by the Ethical Committee of the Clalit Health Services, located at the Soroka Medical Center, Beer-Sheva, Israel. Written informed consent to participate in this study was provided by the participants' legal guardian/next of kin.

## Author Contributions

NB analyzed the data. AW and DM drafted the manuscript. NB, MA, YS, DC, AC, and HA reviewed and critically revised the paper. All authors conceived the experiment.

### Conflict of Interest

The authors declare that the research was conducted in the absence of any commercial or financial relationships that could be construed as a potential conflict of interest.
